# Co-Based Nanosheets with Transitional Metal Doping for Oxygen Evolution Reaction

**DOI:** 10.3390/nano12111788

**Published:** 2022-05-24

**Authors:** Chunhua Xiong, Chao Cai

**Affiliations:** 1 College of Air Traffic Management, Civil Aviation Flight University of China, Guanghan 618307, China; xch@cafuc.edu.cn; 2 School of Physics, University of Electronic Science and Technology of China, Chengdu 610054, China

**Keywords:** transition metal-based materials, two-dimension nanosheets, amorphous materials, electrocatalysts, low bonded oxygen

## Abstract

Activated two-dimension (2D) materials are used in various applications as high-performance catalysts. Breaking the long-range order of the basal plane of 2D materials can highly promote catalytic activity by supplying more active sites. Here we developed a method to synthesize ultrathin MCoO_x_ (M = V, Mn, Fe, Ni, Cu, Zn) amorphous nanosheets (ANSs). These Co-based ANSs show high oxygen evolution reaction (OER) activity in alkaline solution due to the broken long-range order and the presence of abundant low bonded O on the basal plane. The stable Fe_1_Co_1_O_x_ ANSs also show an overpotential of ca. 240 mV of achieving 10 mA/cm^2^ in OER, better than most reported transition metal-based electrocatalysts.

## 1. Introduction

As a fundamental electrochemical half-reaction, oxygen evolution reaction (OER) is involved in various important energy-related technological applications, such as water splitting [1] and Zn-air batteries [2]. However, the sluggish dynamic of the four electron process in OER has highly hindered the development of the oxygen-involved reaction. The relevant reaction process is shown below [3].

(1) $${\mathrm{OH}}^{-}+*↔{\mathrm{OH}}^{*}+e^{-}$$

(2) $${\mathrm{OH}}^{-}+{\mathrm{OH}}^{*}↔O^{*}+H_{2}O+e^{-}$$

(3) $$O^{*}+{\mathrm{OH}}^{-}↔{\mathrm{OOH}}^{*}+e^{-}$$

(4) $${\mathrm{OOH}}^{-}+{\mathrm{OH}}^{-}↔*+O_{2}+H_{2}O+e^{-}$$

Currently demonstrated electrocatalysts for OER are Ru/Ir-based materials [4], which are costly to realize the practical applications. Developing low-cost, efficient electrocatalysts is a potential approach to meet the demand of various applications [5,6]. Transition metal-based catalysts are widely investigated for OER, including oxides [5], phosphides [7], selenides [8], etc. Co-based nanostructures show high catalytic activity and stability in OER, demonstrated by numerous investigations [9,10,11]. Unfortunately, these emerging catalysts still suffer insufficient development, especially in those novel and facile approaches for preparing desired electrocatalysts of such materials with well-defined nanostructures. Therefore, exploring new methods for synthesizing uniform nanomaterials is significant for a wide range of applications and for developing modern synthetic methodology.

Two-dimension (2D) materials are widely used as electrocatalysts because of their high surface area for contacting electrolytes [12], the low energy barrier for mediums absorption [13], and the Fermi-level nearby density of states that resemble noble metals [14]. However, the active sites for catalytic applications are usually located at the edge and defective sites, such as MoS2 [15], WS2 [16], WSe2 [17], etc. Therefore, the basal plane activation is a primary issue in generating efficient catalysts based on 2D materials. The current methods to activate the basal plane are localized area modification, such as manufacturing defects [15], introducing alien species [18], and partially oxidizing atoms on the basal plane [19]. However, realizing large area activation on 2D materials still is a challenge to obtain desired catalysts. Breaking the long-range order can be an efficient way to generate more active sites or realize the large area activation on 2D materials [20]. For instance, the amorphous FeCo-hydroxide nanosheets (NSs) can obtain more active sites by modifying the OH^−^ on the basal plane [21]. Meanwhile, breaking the long-range order of FePO4 also can achieve better OER activity than the crystallized one [22]. Moreover, this activation method also can be used to manipulate the crystal/amorphous feature of FeCoPO4 to obtain a higher activity of hydrogen evolution reaction [23]. Therefore, exploring the general approach to synthesizing amorphous 2D materials could be an emerging point for developing efficient catalysts.

**Scheme 1:**
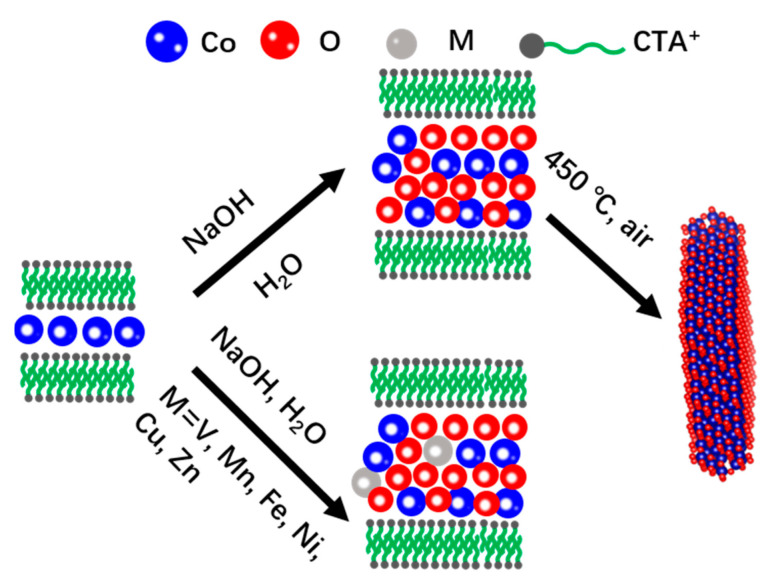
The growth process of CoO-based ANSs. This method also can be used to synthesize NiO ANSs with Ni substituted Co in the first step.

Co-based materials are widely used for OER due to their high intrinsic activity, processability at the atomic scale, and low-cost [24,25]. Albeit many strategies have been developed to generate Co-based materials, the common methods cannot generate amorphous nanosheets, such as the hydrothermal method, [26] sol–gel method [27], chemical vapor method [28], etc. In this work, we synthesized various Co-based amorphous nanosheets (ANSs) using the coprecipitation method in a cetyltrimethylammonium bromide (CTAB) solution. We demonstrate the sheet-like morphology of Co-based materials is remained after introducing alien species, including V, Mn, Fe, Ni, Cu, and Zn oxides (Scheme 1). The thickness of CoOx and Fe_1_Co_1_O_x_ ANSs is only ca. 1 nm. This method can also be used to synthesize ultrathin NiOx ANSs. The Fe doping in CoOx NSs leads to the oxidation process of Co3+ to Co4+ in OER. CoOx ANSs show higher OER activity than the crystallized Co3O4 NSs, demonstrating that low bonded oxygen can also promote the OER. CoO-based materials show high activity in OER, with the overpotential of ca. 240 mV for achieving 10 mA/cm^2^ (Fe_1_Co_1_O_x_ ANSs).

## 2. Materials and Methods

We concluded the growth process of CoO-based ANSs, as shown in Scheme 1. Details of synthesis procedure can be found in supporting materials. The Powder X-ray diffraction (XRD) pattern shows the amorphous feature of CoOx ANSs (Figure S1). MCoOx (M = V, Mn, Fe, Ni, Cu, and Zn) ANSs are synthesized by using M/Co molar ratios of 1:1. The amorphous feature remains after doping Fe, being verified by XRD (Figure S1). The Fe, Co, and O show homogeneous distribution being demonstrated by the energy dispersive spectroscopy (EDS) method (Figure S2). The 2D morphologies of MCoOx ANSs are checked by scanning electron microscopy (SEM) (Figure S3). For comparison, crystallized Co3O4 NSs are prepared by annealing CoOx ANSs. Peaks in XRD pattern of Co_3_O_4_ NSs are consistent with JCPDS No. 43-1003 (Figure S4).

## 3. Results

### 3.1. Electrochemical Performance

**Figure 1:**
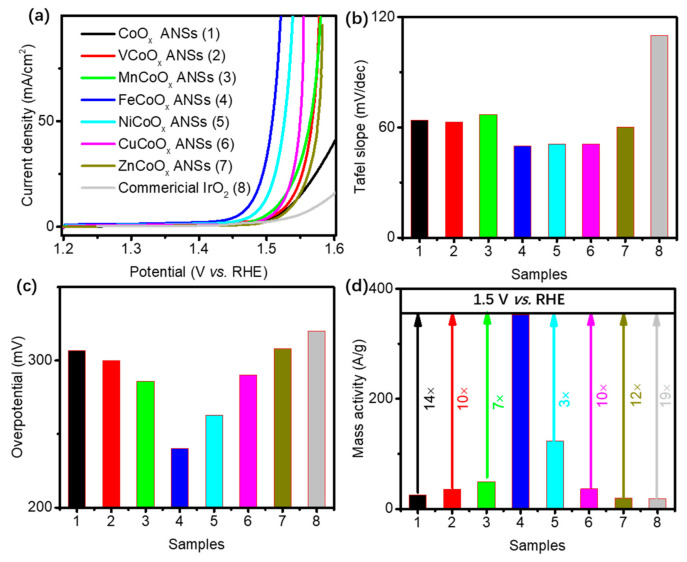
Electrochemical characterization of MCoO_x_ ANSs. Polarization curves (**a**), specific Tafel slopes (**b**), specific overpotential of achieving 10 mA/cm^2^ (**c**), and specific mass activity at 1.5 V vs. RHE (**d**) of Co-based ANSs. Fe_1_Co_1_O_x_ ANSs show highest OER activity among these Co-based ANSs.

The catalytic activity of various Co-based ANSs is tested in a polytetrafluoroethylene (PTFE) bottle. We used Co-based ANSs as electrocatalysts for OER in 1 M KOH at room temperature (current density in this work is normalized by the geometric area of the used electrode). To begin with, we demonstrated that CoO_x_ ANSs show better OER performance than Co_3_O_4_ NSs (Figure S5). This result indicates that breaking the long-range order on 2D materials can activate the basal plane for catalytic activity [20]. The electrochemical surface area (ECSA) value is positively related to the number of active sites. The CoO_x_ ANSs possess an ECSA of 4-fold of Co_3_O_4_ NSs (Figure S6), demonstrating the highly increased active site numbers in CoO_x_ ANSs. Figure 1a plots the polarization curves of Co-based ANSs. These MCoO_x_ ANSs show better OER activity than commercial IrO_2,_ comparing the overpotential of achieving a current density of 10 mA/cm^2^ (Table S1). Small Tafel slope benefits the practical applications, where the highly increased current density needs only a low potential increase. The Fe_1_Co_1_O_x_ ANSs show the lowest Tafel slope (ca. 50 mV/dec) value among those prepared MCoO_x_ ANSs (Figure 1b) (Table S1). Figure 1c summarizes the overpotential of achieving 10 mA/cm^2^ of MCoO_x_ ANSs and commercial IrO_2_. Fe_1_Co_1_O_x_ ANSs possess an overpotential of ca. 240 mV, which is better than other MCoO_x_ ANSs (Table S1) and the state-of-art Co-based OER catalysts (Table S2) [29,30,31,32,33,34,35,36,37,38,39,40]. The higher mass activity shows the lower cost of OER catalysts in practical applications. As shown in Figure 1d, The Fe_1_Co_1_O_x_ ANSs show the highest mass activity of ca. 353 A/g, which is ca. 19-fold of commercial IrO_2_ (ca. 18.53 A/g). Fe_1_Co_1_O_x_ ANSs also show good activity and structural stability in OER at 1.5 V vs. RHE (Figure S7). Compared to the stability test of Co-based NSs (Figure S5), we concluded that Co could be responsible for the high stability of OER activity. Indeed, these results demonstrate the highly promising Fe_1_Co_1_O_x_ ANSs as practical catalysts for OER.

### 3.2. Structural Characterization

**Figure 2:**
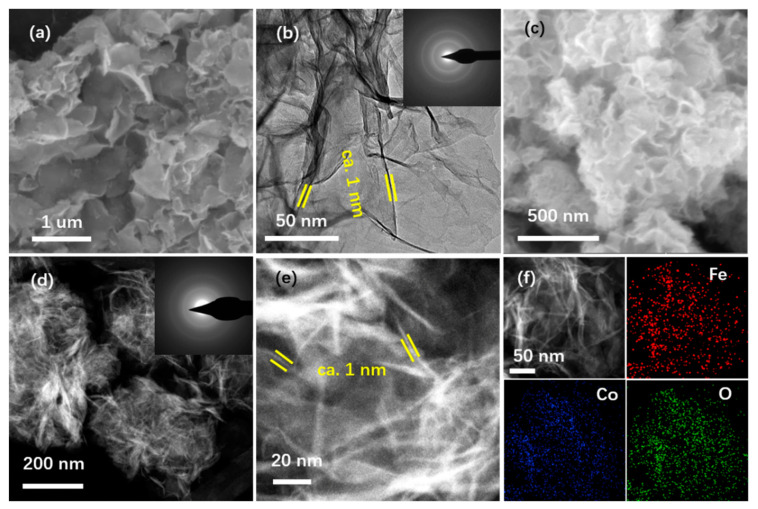
Structural characterization of CoO_x_ ANSs and Fe_1_Co_1_O_x_ ANSs. SEM image (**a**) and TEM image of CoO_x_ ANSs (**b**). Inset in (**b**) is SAED pattern of CoO_x_ ANSs. SEM image (**c**), low magnification HAADF image (**d**), high magnification image (**e**), EDS mapping (**f**) of Fe_1_Co_1_O_x_ ANSs. Inset in (**d**) is SAED pattern of Fe_1_Co_1_O_x_ ANSs. The CoO ANSs and Fe_1_Co_1_O_x_ ANSs possess similar thickness of ca. 1 nm.

The structural characterizations focus on the Fe_1_Co_1_O_x_ ANSs because Fe_1_Co_1_O_x_ ANSs show the best OER activity (Figure 1). As shown in Figure 2, we use SEM and transmission electron microscopy (TEM) method to character ANSs. SEM and TEM images show that CoO_x_ ANSs have well-defined 2D structures (Figure 2a,b). The selected area electron diffraction (SAED) pattern, inset in Figure 2b, certifies the amorphous feature of CoO_x_ ANSs again. Figure 2b shows that CoO_x_ ANSs possess a thickness of ca. 1 nm. The SEM and low magnification high angle annular dark field (HAADF) images (Figure 2c,d) show the hierarchical nanostructure of Fe_1_Co_1_O_x_ ANSs, with a width of ca. 400 nm. A high-resolution HAADF image verifies the Fe_1_Co_1_O_x_ ANSs thickness of ca. 1 nm, as shown in Figure 2e. EDS mapping shows the homogeneous distribution of Fe, Co, and O (Figure 2f). The ultrathin Co-based ANSs formation can be attributed to the surfactant of CTAB (with a high concentration of 0.05 M) and NaOH (with fast precipitation rate in solution, formation of amorphous MCoO_x_ ANSs) [41]. The concentration of CTAB is above 2-fold its second micelle concentration (0.021 M) [42], leading to the CTAB micelles in reaction solution tending to form two layer structures (Scheme 1). The ultrathin layer structure and elemental homogeneous distribution of Fe_1_Co_1_O_x_ ANSs may be caused by the fast nuclei of Fe and Co in alkaline solution during the coprecipitation process. This growth mechanism also works in synthesizing NiO_x_ ANSs (Figures S8 and S9).

## 4. Discussion

### 4.1. Surface Chemistry

**Figure 3:**
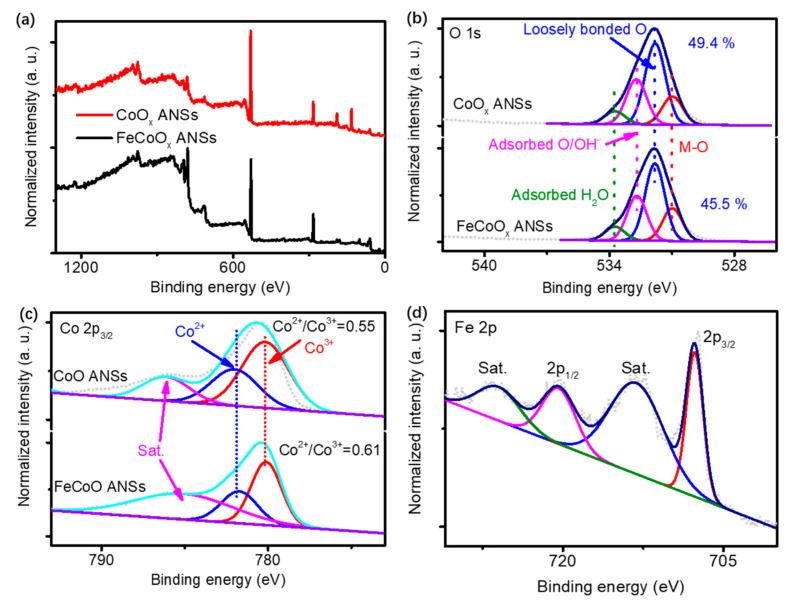
XPS spectrum of CoO_x_ ANSs and Fe_1_Co_1_O_x_ ANSs. Survey spectrum (**a**), O 1s spectrum (**b**), Co 2p spectrum of CoO_x_ ANSs and FeCoO_x_ ANSs (**c**), and Fe 2p spectrum of FeCoO_x_ ANSs (**d**). The Fe loading on CoO_x_ ANSs makes the change of Co 2p peaks, verifying the strong synergistic effect between Fe and Co oxides in 2D structures.

The strong synergistic effect between cation and anion in FeCo-based nanocrystals is usually used to generate highly active OER electrocatalysts [43,44]. Herein, X-ray photoelectron spectrum (XPS) is used to check the electronic configuration and O state of CoO_x_ ANSs and Fe_1_Co_1_O_x_ ANSs. Figure 3a shows the survey spectrum of CoO_x_ ANSs and Fe_1_Co_1_O_x_ ANSs. Figure 3b plots the fitted O 1s spectrum. The three characteristic peaks of O 1s are consistent with oxygen atoms bound to metal atoms (530.9 eV), low bonded oxygen (531.8 eV), OH^−^ or surface-adsorbed oxygen (532.7 eV), and water molecules on the surface (533.74 eV) [45]. The CoO_x_ ANSs have a low bonded oxygen ratio of 49.4%, higher than 18.0% of Co_3_O_4_ NSs (Figure S10), demonstrating that the broken long-range order of ANSs leads to a high ratio of O in NSs. Moreover, extra Fe doping leads to the larger low bonded O ratio of 45.5% in Fe_1_Co_1_O_x_ ANSs, because Fe can modify the adsorption state of surrounded Co. To clarify this, we analyze the state variations of Co after Fe doping. Figure 3c summarizes the fitted Co 2p spectrum of CoO_x_ ANSs and Fe_1_Co_1_O_x_ ANSs. The peaks located at ca. 796.7 eV and ca. 780.9 eV correspond to Co 2p_1/2_ and Co 2p_3/2_ of Co^3+^, respectively [29]. The peaks of Co^2+^ appear at ca. 781.1 eV (CoO_x_ ANSs) to ca. 781.8 eV (Fe_1_Co_1_O_x_ ANSs). The Co^2+^/Co^3+^ ratio in Fe_1_Co_1_O_x_ ANSs (0.61) is higher than 0.55 in CoO_x_ ANSs. The high Co^2+^ ratio in Fe_1_Co_1_O_x_ ANSs can be due to the extra Fe bonds on some O near the Co center. Figure 3d shows the fitted Fe 2p spectrum. The peaks located at ca. 720.5 eV and ca. 707.7 eV are consistent with 2p_1/2_ and 2p_3/2_ of Fe^3+^, respectively [46]. Therefore, the Co modulation is determined by the Fe^3+^, leading to the OER activity increase.

### 4.2. Reaction Process

**Figure 4:**
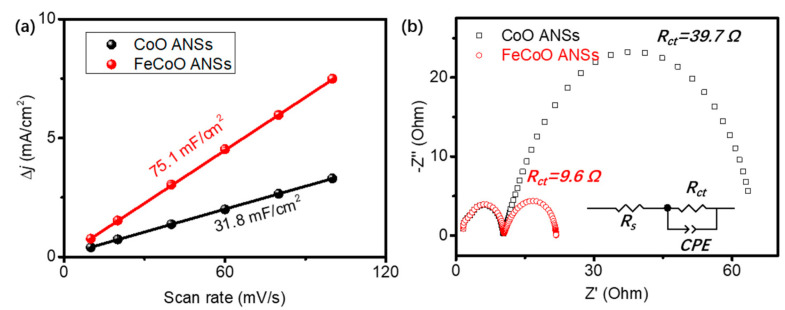
Analysis of catalysts and electrode. ECSA (**a**) and Nyquist plots (**b**) of ANS. The Fe doping can effectively promote the reactive sites with ca. 2-fold (**a**). The charge transfer efficiency between glassy carbon support and FeCoO_x_ ANSs is very low (**b**). ECSA is calculated from double layered capacitance (*C_dl_*) at 1.15 V vs. RHE.

The high valance Co ions are considered the active sites for OER at a high potential of 1.24 to 1.54 V vs. RHE (Co^3+^ and Co^4+^) [47]. The intrinsic activity of Co-based materials is usually determined by the Co^3+^ and Co^4+^ formation during OER. The abundant low bonded oxygen can promote the high Co cations formation; Refs. [48,49] meanwhile lead to the formation of delocalized electrons in Co, and thus promote the adsorption efficiency of water molecules on Co cations and enhance conductively and catalytic activity [50,51,52]. Furthermore, the low bonded O in CoO_x_ ANSs can be regarded as pre-activated O^2−^ or O^0^, which can be preferentially oxidized or released during OER and thus promote the OER [53]. These appearances are demonstrated by the fact that low bonded O-rich CoO_x_ ANSs have higher OER activity than Co_3_O_4_ NSs (Figures S5 and S6). Moreover, the Fe can promote the OER activity of Co by decreasing the intermediate formation energy [54], the adsorption energy of OH^−^, and the desorption efficiency of O during OER [55]. This feature can lead to an ECSA value increase [46]. Figure 4a shows that Fe_1_Co_1_O_x_ ANSs possess the ECSA value of ca. 75.1 mF/cm^2^, which is 2-fold of CoO_x_ ANSs (ca. 31.8 mF/cm^2^). Moreover, the changed electronic state on Co also can promote the conductivity, which shows an impact on efficacious charge transfer between support and catalysts [1,48]. The charge transfer resistance (*R_ct_*) is analyzed by electrochemical impedance spectrum (EIS) (measured at 1.53 V vs. RHE), as shown in Figure 4b. The *R_ct_* of Fe_1_Co_1_O_x_ ANSs is ca. 9.6 Ω, which is smaller than 39.7 Ω of CoO_x_ ANSs, suggesting an accelerated charge transfer coefficient between FeCoO_x_ ANSs and support. Indeed, the outstanding OER activity of FeCoO_x_ ANSs may originate from the abundant low bonded O, intrinsic activity of Co, which is highly activated by Fe^3+^.

### 4.3. Fe Doping Amount

As the above results show, Fe plays an important role in determining the OER activity of Fe_x_Co_1−x_O_y_ ANSs. The OER activity is amount doping dependent in synergistic hybrids. The Fe_x_Co_1−x_O_y_ ANSs with different Fe atom doping show similar onset potential in OER (around 1.42 V vs. RHE) (Figure S11), far lower than the one of 1.49 V vs. RHE in CoO_x_ ANSs (70 mV). Moreover, Figure S5a shows that the onset potential of Co_3_O_4_ NSs is 1.52 V vs. RHE, lower than the one in CoO_x_ ANSs (30 mV). These results indicate that the OER rate and characteristic activity are major determined by the Fe but not the low bonded oxygen in Fe_1_Co_1_O_x_ ANSs. After Fe doping, the adsorption energy state of these negatively charged species on the Co^3+^/Co^4+^ delocalized center can be different, which is sensitive to the difference in the electron affinity between Fe (15 kJ/mol) and Co (63 kJ/mol) [48]. The high electron affinity of Co ensures the high concentration of negatively charged species around Co cations with low bonded or activated O surrounded, with accelerated Co^3+^/Co^4+^ formation during OER [49]. The concentrated negatively charged species around Co in Fe_x_Co_1-x_O_y_ ANSs also relates to the Fe doping ratio in the ANS’s system [6,56,57,58,59]. Fe_x_Co_1-x_O_y_ ANSs with different Fe molar ratio doping show modified charge transport efficiency (Figure S11b). Fe_1_Co_1_O_x_ ANSs show the lowest Tafel slope of 50 mV/dec, verifying the highest adsorption/desorption efficiency on delocalized Co cations center with low bonded or activated O surrounded in OER. By further increasing the Fe doping to 0.8 mmol, the Tafel slope increases (67 mV/dec) because Fe oxides itself is not good OER catalysts. Therefore, optimization of Fe doping in Fe_1_Co_1_O_x_ ANSs determines the OER activity by changing the absorption/desorption on Co^3+^/Co^4+^ cations surrounded by low bonded O and activated O.

## 5. Conclusions

In conclusion, we developed a general approach for preparing ultrathin Co-based ANSs with abundant low bonded oxygen. The intrinsic OER activity of Co-based ANSs can be modified by introducing alien species, where the Fe doped Co NSs possess the highest OER activity. Such superior OER activity of Fe_1_Co_1_O_x_ ANSs benefits from the promoted oxidation process of Co^3+^ to Co^4+^, high intrinsic catalytic activity, and large electrochemically active surface area. We believe that this work developed a potential way to generate highly efficient catalysts based on activated 2D materials.

## Data Availability

Not applicable.

## Supplementary Materials

The following supporting information can be downloaded at: https://www.mdpi.com/article/10.3390/nano12111788/s1, Figure S1: XRD pattern of as-prepared CoO_x_ and Fe_1_Co_1_O_x_ NSs; Figure S2: EDS mapping of Fe_1_Co_1_O_x_ ANSs in SEM modes; Figure S3: SEM images of MCoO_x_ NSs. These NSs are based on Co, with M: Co molar ratio of 1:1 in solution; Figure S4: XRD pattern and SEM image of Co_3_O_4_ NSs. Because of the thermal relaxation, the annealed Co_3_O_4_ NSs shows good crystalline. SEM image clarifies the 2D feature of Co_3_O_4_ NSs (Figure S4b); Figure S5: Electrochemical characterization of CoO_x_ ANSs and Co_3_O_4_ NSs. Polarization curves (a), Tafel slopes (b), stability test of CoO ANSs (c), and stability test of Co_3_O_4_ NSs (d); Figure S6: ECSA of CoO_x_ ANSs and Co_3_O_4_ NSs. *C_dl_* is tested from 1.15 V vs. RHE; Figure S7: Accelerating degradation of Fe_1_Co_1_O_x_ ANSs in OER. Stability test of Fe_1_Co_1_O_x_ ANSs (a) and structure of FeCoO_x_ ANSs after use (b) in alkaline solution. The collective electron transport (a) and sustained 2D feature (b) in OER demonstrate the high stability of FeCoO_x_ ANSs for adapting long-time oxidizing condition; Figure S8: SAED and TEM images of NiO_x_ ANSs. The NiO_x_ ANSs also show ultrathin 2D feature. In contrast, the width of NiO_x_ ANSs (ca. 200 nm) is much smaller than CoO_x_ ANSs (>1 um); Figure S9: EELS of NiO_x_ ANSs. The EELS spectrum demonstrates that the Ni-based NSs prepared in CTAB solution is composited by Ni and O; Figure S10: XPS spectrum of Co 2p and O1s of Co_3_O_4_ NSs; Figure S11: Electrochemical test of Fe_x_Co_1-x_O_y_ ANSs with different Fe feeding in preparing step. The Co feed amount in these materials is 0.4 mmol; Table S1: Parameter of electrocatalysts for OER in 1 M KOH; Table S2: Collection of reported Co-based electrocatalysts on glassy carbon electrode for OER in 1 M KOH. References [29,30,31,32,33,34,35,36,37,38,39,40] are cited in the supplementary materials.
