# Supplementary material for: Co-Based Nanosheets with Transitional Metal Doping for Oxygen Evolution Reaction
Source: Nanomaterials (Basel). 2022 May 24;12(11):1788. doi: 10.3390/nano12111788 (PMC9182512; doi:10.3390/nano12111788)
Supplement: Supplementary file 1 [file nanomaterials-12-01788-s001.zip › nanomaterials-1665989-SI.pdf]

Supplementary Materials

Co-Based Nanosheets with Transitional Metal Doping for Oxygen Evolution Reaction

Chunhua Xiong [1] and Chao Cai [2,*]

[1] College of Air Traffic Management, Civil Aviation Flight University of China, Guanghan 618307, China; xch@cafuc.edu.cn
[2] School of Physics, University of Electronic Science and Technology of China, Chengdu 610054, China
* Correspondence: zchaotsai@163.com

Chemicals

VCl_4 (>98 %), $MnSO_4$ (>98 %), $Fe(NO_3)_3{\cdot}6H_2O$ (>98 %), $Co(NO_3)_2{\cdot}6H_2O$ (>98 %), $Ni(NO_3)_2{\cdot}6H_2O$ (>98 %), $CuCl_2{\cdot}2H_2O$ (>97 %), $ZnSO_4$ (>98 %), Sodium borohydride (SB) (>98 %), CTAB (>95 %), ethanol (>99 %), acetone (>97 %), potassium hydroxide (99.999 %), and isopropanol (>99 %) are purchased from Aladdin in Shanghai, China. Nafion solution (4.5 wt. %) is purchased from sigma, which is provided by The Chemours Company FC, LLC. Deionized water is 18.2 Ω/cm.

Synthesis of CoO_x ANSs

In air, 0.4 mmol $Co(NO_3)_2{\cdot}6H_2O$ and 1 mmol CTAB are dissolved into 20 mL water with stirring speed of 600 rpm, forming pink solution. Then 10 mL NaOH solution (0.5 M) and 10 mL SB solution (0.1 mM) are added into pink solution quickly at room temperature. After further stirring of 30 min, the green products are washed 2 times by water and 3 times by ethanol/acetone (1:1) to remove surfactant, dried in air at 80 ℃ for 1 day.

Synthesis of $MCoO_x$ ANSs

The preparation and wash processes of $MCoO_x$ ANSs are similar to the CoO_x ANSs with extra metal salt added into pink solution (M/Co=1:1). The Fe/Co ratio is controlled via change the feed amount of $Fe(NO_3)_3·6H_2O$.

Synthesis of NiO_x ANSs

The preparation and wash processes of NiO_x ANSs are similar to the CoO_x ANSs by adding 0.4 mmol $Ni(NO_3)_2·6H_2O$ instead.

Crystal Co_3O_4 NSs are prepared by annealing prepared CoO_x ANSs in air at 450 ℃ for 30 min with a heating rate of 10 ℃/min.

X-ray diffraction (XRD) (Bruker ECO D8 power X-ray diffractometer with Cu Kα radiation) were utilized to determine the crystal structure of the samples.

The morphology and microstructure characterizations of the as-prepared samples were investigated by ***scanning electron microscopy (SEM)*** (FEI, Helios Nanolab 600i, 2kV), ***Transmission electron microscopy (TEM)*** and selected area electron diffraction (SAED) (FEI, ETEM, G2, 200kV), ***scanning transmission electron microscopy with an energy dispersive X-ray spectroscopy attachment*** (STEM-EDS, FEI Titan Themis, 300 kV).

XPS measurements were conducted on the Axis Ultra DLD multitechnique surface analysis system and relative curves were calculated by C1s (284.8 eV).

Electrochemical characterization

All the experiments are carried out in air at room temperature. Prepared samples loading on carbon with mass containing of 20 wt. %. Isopropanol, water and Nafion solution (volume ratio is 1:3:0.005) is used as solution for

preparing catalyst ink (2 mg/mL). Amount of ink is cased onto a polished glassy carbon electrode as working electrode, with catalyst loading of ca. 0.113 mg/cm^2. In a three-electrode system, 1 M KOH is used as electrolyte, Ag/AgCl and graphite rod are used as reference electrode and counter electrode respectively. Before test, the working electrode is immersed into electrolyte for 2 h. Line scan voltammetry (0.002 V/s on a 1600 rpm RDE) is used to character the catalytic performance. C_{dl} is calculated from the difference between anodic and cathodic current density at 1.15 V *vs.* RHE (relative cyclic voltammetry is carried out from 1.1-1.2 V *vs.* RHE).

All the experimental equipment except TEM is located at the University of Electronic Science and Technology of China, Chengdu 610054, China. The TEM was conducted at Southern University of Science and Technology in Shenzhen 518000, China.

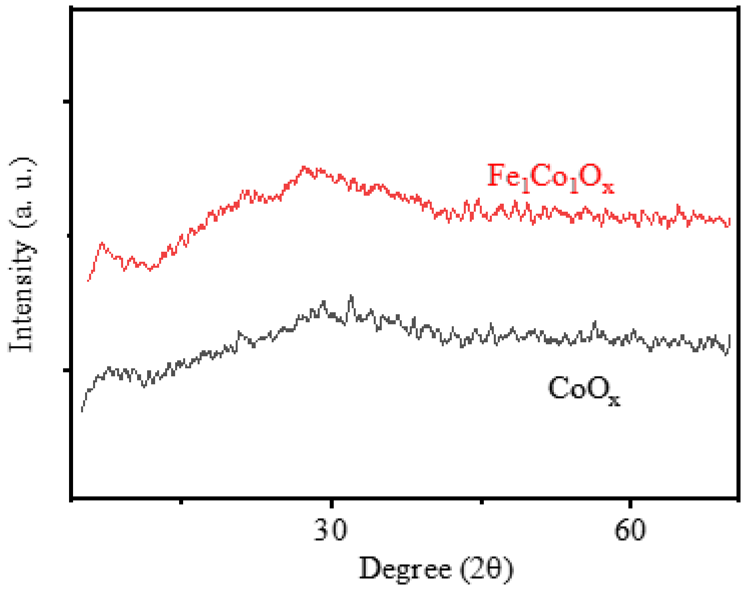

Figure S1. XRD pattern of as-prepared CoO_x and $Fe_1Co_1O_x$ NSs.

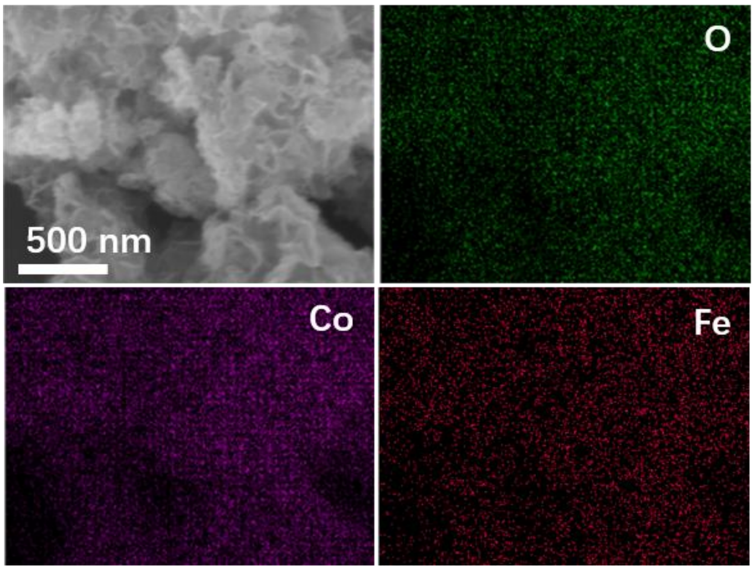

Figure S2. EDS mapping of $Fe_1Co_1O_x$ ANSs in SEM modes.

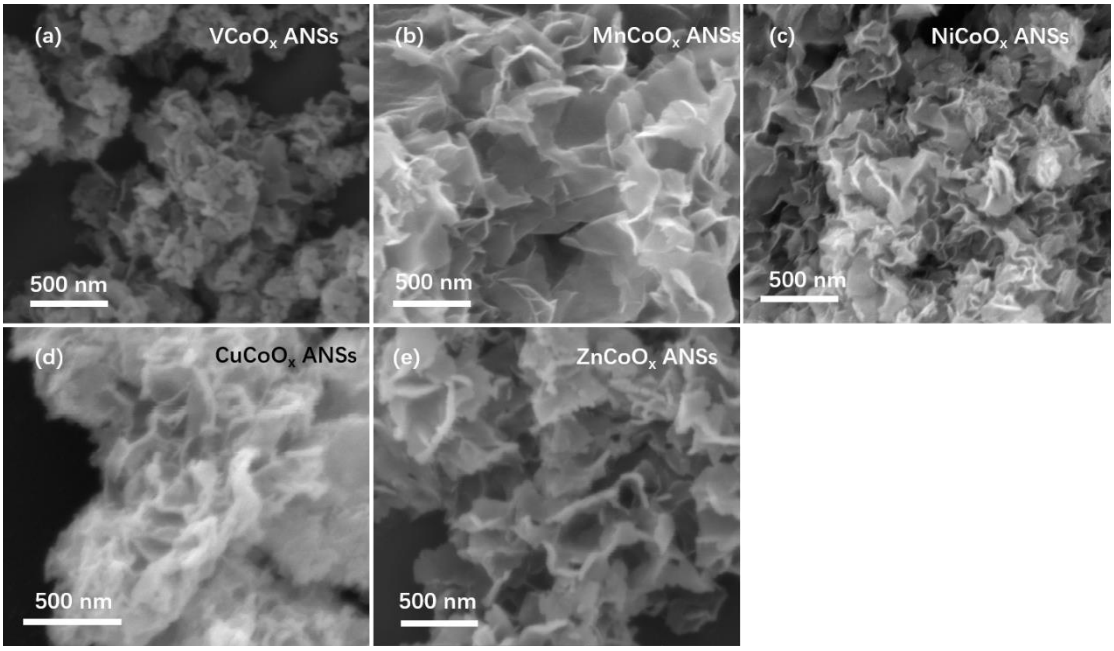

Figure S3. SEM images of $MCoO_x$ NSs. These NSs are based on Co, with M:Co molar ratio of 1:1 in solution. All the acquired materials possesses sheet-like feature.

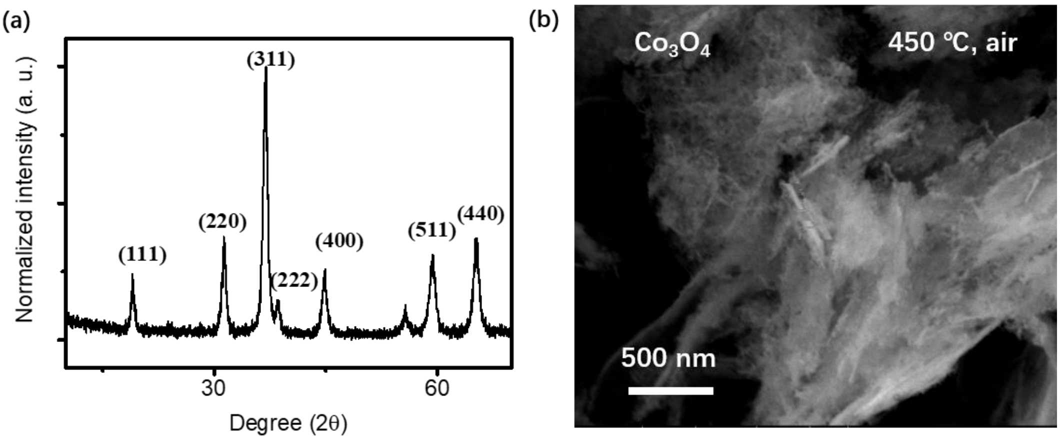

Figure S4. XRD pattern and SEM image of Co_3O_4 NSs. The materials after annealing possess good crystalline, in line with JCPDS #42-1467. Because of the thermal relaxation, the annealed Co_3O_4 NSs shows good crystalline. SEM image clarifies the 2D feature of Co_3O_4 NSs (Figure S4b).

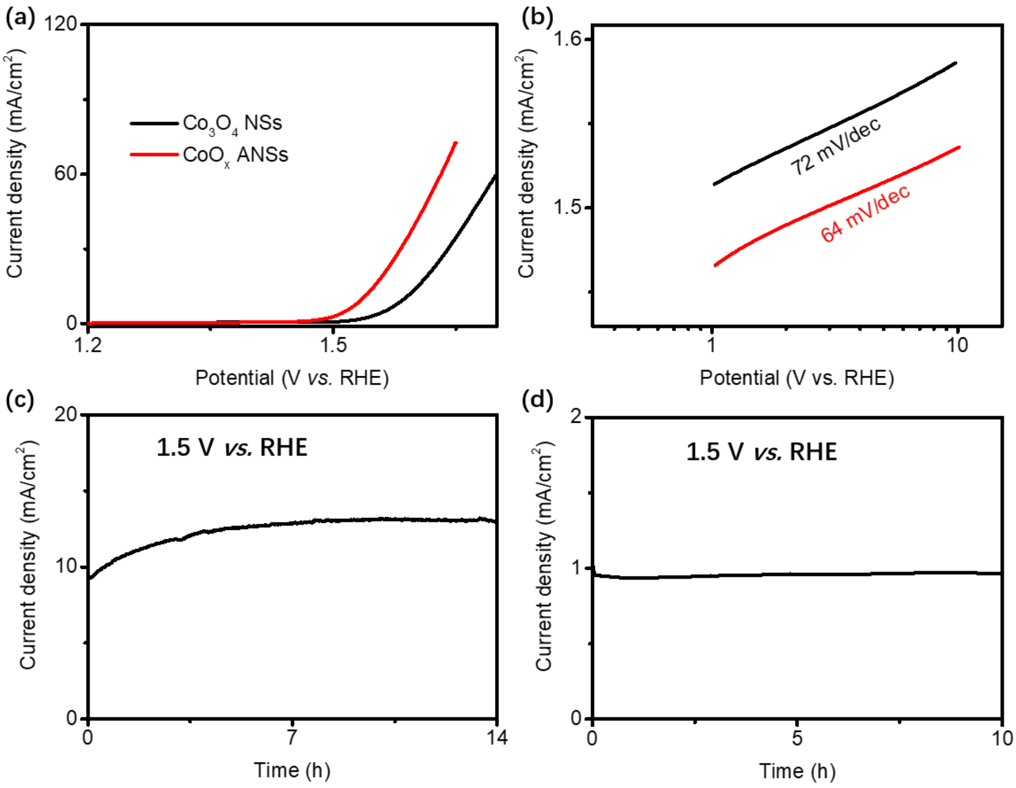

Figure S5. Electrochemical characterization of CoO_x ANSs and Co_3O_4 NSs. Polarization curves (a), Tafel slopes (b), stability test of CoO ANSs (c), and stability test of Co_3O_4 NSs (d).

CoO_x ANSs show higher OER activity, which is ca. 3.6-fold of Co_3O_4 NSs [calculated from specific current density at 1.5 V *vs.* RHE)]. CoO_x ANSs also have lower Tafel slope (64 mV/dec) than Co_3O_4 NSs (72 mV/dec). Both Co_3O_4 NSs and CoO_x ANSs show good stability in OER at 1.5 V *vs.* RHE, indicating the priority of 2D feature of CoO-based materials for efficient OER catalysts.

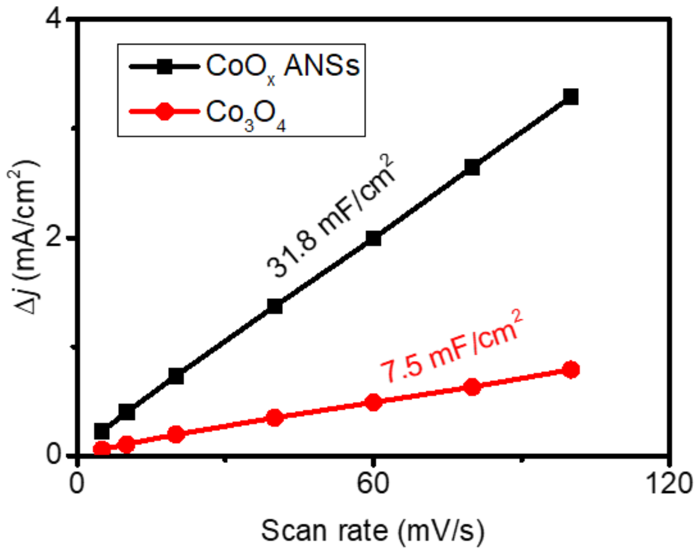

Figure S6. ECSA of CoO_x ANSs and Co_3O_4 NSs. C_{dl} is tested from 1.15 V *vs.* RHE.

Table S1. Parameter of electrocatalysts for OER in 1 M KOH.

Sample	Tafel slope (mV/dec)	Overpotential at 10 mA/cm^2 (mV)	Mass activity at 1.5 V *vs.* RHE (A/g)
Co_3O_4 NSs	72	357	8.2
CoO_x ANSs	64	307	25.6
$VCoO_x$ ANSs	63	300	35.3
$MnCoO_x$ ANSs	67	286	49.4
**$Fe_1Co_1O_x$ ANSs**	**50**	**240**	**353.0**
$NiCoO_x$ ANSs	51	263	123.6
$CuCoO_x$ ANSs	51	290	36.2
$ZnCoO_x$ ANSs	60	308	19.4
**Commercial IrO_2**	**70**	**320**	**18.5**
Commercial IrO_2 in Ref. [1]	52	330	27.5 @ 1.53 V *vs.* RHE

$MCoO_x$ ANSs have the atomic ratio of M/Co=1:1.

Table S2. Collection of reported Co-based electrocatalysts on glassy carbon electrode for OER in 1 M KOH.

Sample	Tafel slope (mV/dec)	Overpotential at 10 mA/cm^2 (mV)	Mass activity (A/g)	Ref.
CoOOH	38	300	66.6 @ 1.53 V vs. RHE	[1]
$CoSe_2$	50	430	--	[2]
Au@Co_3O_4	60	370	--	[3]
NiO/CoN	35	300	853 @ 1.596 V vs. RHE	[4]
NG-$CoSe_2$	40	366	63.5 @ 1.596 V vs. RHE	[5]
CoFe LDH	34	264	--	[6]
CoNi-P	52	270	--	[7]
rGO@CoFe-Pi	36	300	160 @ 1.59 V vs. RHE	[8]
CoNi-P	84	292	253 @ 1.59 V vs. RHE	[9]
Co_3O_4	58	367	--	[10]
Ir@Co	90	273	--	[11]
Co/Mo_2C@CNTs	90	356	--	[12]

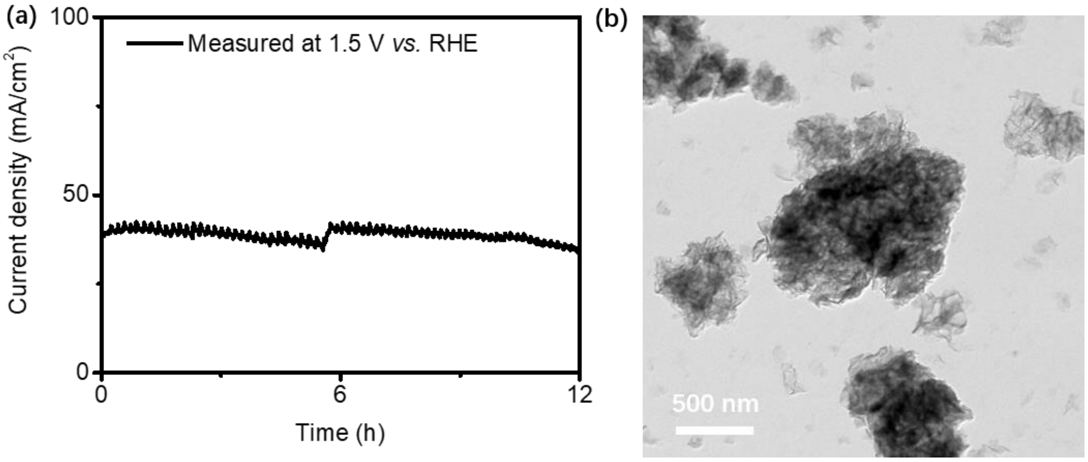

Figure S7. Accelerating degradation of $Fe_1Co_1O_x$ ANSs in OER. Stability test of $Fe_1Co_1O_x$ ANSs (a) and structure of $FeCoO_x$ ANSs after use (b) in alkaline solution. The collective electron transport (a) and sustained 2D feature (b) in OER demonstrate the high stability of $FeCoO_x$ ANSs for adapting long-time oxidizing condition.

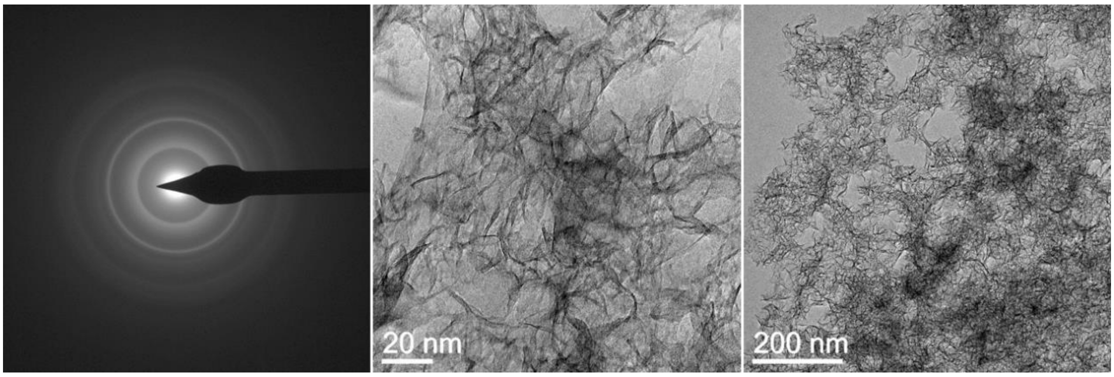

Figure S8. SAED and TEM images of NiO_x ANSs. The NiO_x ANSs also show ultrathin 2D feature. In contrast, the width of NiO_x ANSs (ca. 200 nm) is much smaller than CoO_x ANSs (> 1 μm).

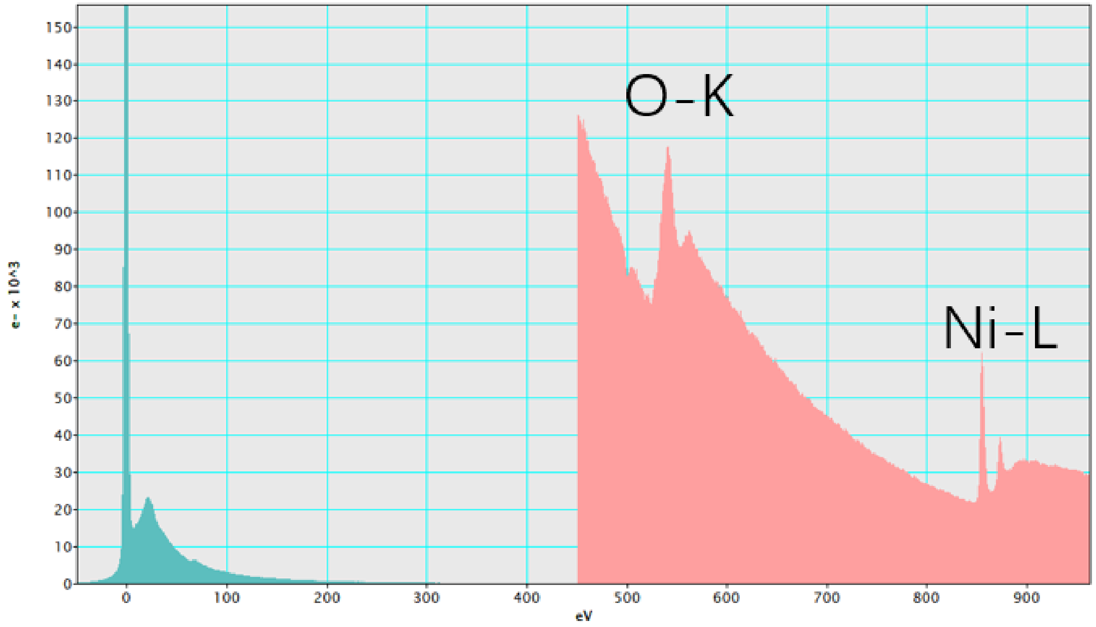

Figure S9. EELS of NiO_x ANSs. The EELS spectrum demonstrates that the Ni-based NSs prepared in CTAB solution is composited by Ni and O.

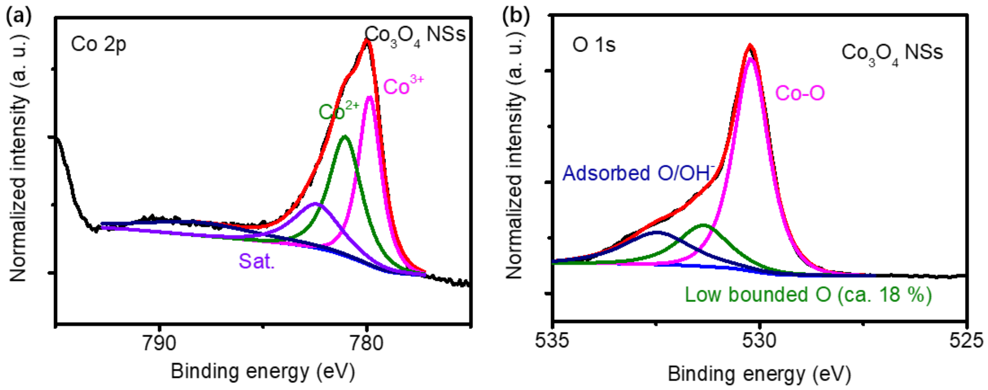

Figure S10. XPS spectrum of Co 2p and O1s of Co_3O_4 NSs.

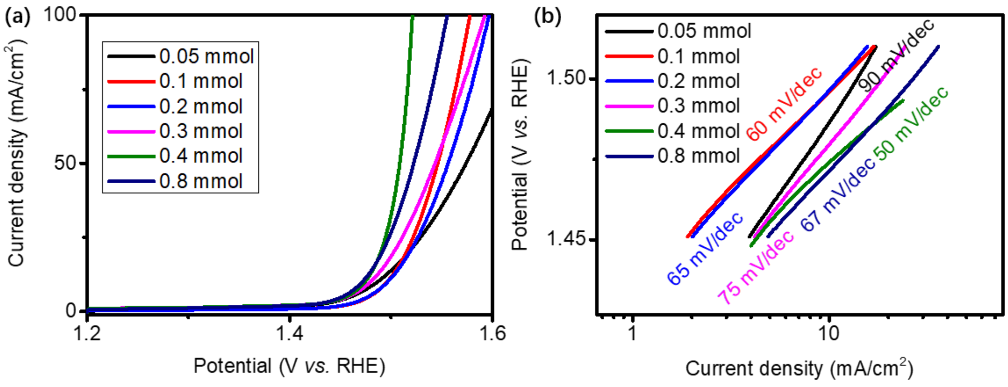

Figure S11. Electrochemical test of $Fe_xCo_{1-x}O_y$ ANSs with different Fe feeding in preparing step. The Co feed amount in these materials is 0.4 mmol.

References and Notes

1. Huang, J., Chen, J., Yao, T., He, J., Jiang, S., Sun, Z., Liu, Q., Cheng, W., Hu, F., Jiang, Y., Pan, Z. and Wei, S., CoOOH Nanosheets with High Mass Activity for Water Oxidation, *Angewandte Chemie*, **2015**, *54*, 8722-8727.

2. Kwak, I. H., Im, H. S., Jang, D. M., Kim, Y. W., Park, K., Lim, Y. R., Cha, E. H. and Park, J., CoSe(2) and NiSe(2) Nanocrystals as Superior Bifunctional Catalysts for Electrochemical and Photoelectrochemical Water Splitting, *ACS applied materials & interfaces*, **2016**, *8*, 5327-5334.

3. Zhuang, Z., Sheng, W. and Yan, Y., Synthesis of Monodispere Au@Co3O4 Core-Shell Nanocrystals and Their Enhanced Catalytic Activity for Oxygen Evolution Reaction, *Advanced Materials*, **2014**, *26*, 3950-3955.

4. Yin, J., Li, Y., Lv, F., Fan, Q., Zhao, Y.-Q., Zhang, Q., Wang, W., Cheng, F., Xi, P. and Guo, S., NiO/CoN Porous Nanowires as Efficient Bifunctional

Catalysts for Zn-Air Batteries, *ACS nano*, **2017**, *11*, 2275-2283.

5. Gao, M. R., Cao, X., Gao, Q., Xu, Y. F., Zheng, Y. R., Jiang, J. and Yu, S. H., Nitrogen-doped graphene supported CoSe(2) nanobelt composite catalyst for efficient water oxidation, *ACS nano*, **2014**, *8*, 3970-3978.

6. Liu, Y., Jin, Z., Li, P., Tian, X., Chen, X. and Xiao, D., Boron- and Iron-Incorporated d oped gr Ultrathin Nanosheets as an Efficient Oxygen Evolution Catalyst, *ChemElectroChem*, **2018**, *5*, 593-597.

7. Xiao, X., He, C.-T., Zhao, S., Li, J., Lin, W., Yuan, Z., Zhang, Q., Wang, S., Dai, L. and Yu, D., A general approach to cobalt-based homobimetallic phosphide ultrathin nanosheets for highly efficient oxygen evolution in alkaline media, *Energy & Environmental Science*, **2017**, *10*, 893-899.

8. Li, J., Zhou, Q., Zhong, C., Li, S., Shen, Z., Pu, J., Liu, J., Zhou, Y., Zhang, H. and Ma, H., (Co/Fe)4O4 Cubane-Containing Nanorings Fabricated by Phosphorylating Cobalt Ferrite for Highly Efficient Oxygen Evolution Reaction, *ACS Catalysis*, **2019**, *9*, 3878-3887.

9. Li, G. L., Zhang, X. B., Zhang, H., Liao, C. Y. and Jiang, G. B., Bottom-up MOF-intermediated synthesis of 3D hierarchical flower-like cobalt-based homobimetallic phophide composed of ultrathin nanosheets for highly efficient oxygen evolution reaction, *Applied Catalysis B-Environmental*, **2019**, *249*, 147-154.

10. Tan, P., Wu, Z., Chen, B., Xu, H. R., Cai, W. Z. and Ni, M., Exploring oxygen electrocatalytic activity and pseudocapacitive behavior of Co3O4 nanoplates in

alkaline solutions, *Electrochimica Acta*, **2019**, *310*, 86-95.

11. Babu, D. D., Huang, Y., Anandhababu, G., Wang, X., Si, R., Wu, M., Li, Q., Wang, Y. and Yao, J., Atomic iridium@cobalt nanosheets for dinuclear tandem water oxidation, *Journal of Materials Chemistry A*, **2019**, *7*, 8376-8383.

12. Ouyang, T., Ye, Y. Q., Wu, C. Y., Xiao, K. and Liu, Z. Q., Heterostructures Composed of N-Doped Carbon Nanotubes Encapsulating Cobalt and beta-Mo2 C Nanoparticles as Bifunctional Electrodes for Water Splitting, *Angewandte Chemie*, **2019**, *58*, 4923-4928.
